# Research progress of ophthalmic preparations of immunosuppressants

**DOI:** 10.1080/10717544.2023.2175925

**Published:** 2023-02-10

**Authors:** Ye Liu, Haonan Xu, Na Yan, Zhan Tang, Qiao Wang

**Affiliations:** a School of Pharmacy, Hangzhou Medical College, Hangzhou, Zhejiang, 310013, China; b Department of Pharmacy, Jin Hua Municipal Maternal and Child Health Care Hospital, Jinhua, Zhejiang, 321000, China; c Key Laboratory of Neuropsychiatric Drug Research of Zhejiang Province, Hangzhou Medical College, Hangzhou, Zhejiang, 310013, China

**Keywords:** Immunosuppressant, ophthalmic, nano-delivery system, immune eye disease

## Abstract

Immune ophthalmopathy is a collection of autoimmune eye diseases. Immunosuppressants are drugs that can inhibit the body’s immune response. Considering drug side effects such as hepatorenal toxicity and the unique structure of the eye, incorporating immunosuppressants into ophthalmic nanodrug delivery systems, such as microparticles, nanoparticles, liposomes, micelles, implants, and *in situ* gels, has the advantages of improving solubility, increasing bioavailability, high eye-target specificity, and reducing side effects. This study reviews recent research and applications of this aspect to provide a reference for the development of an ophthalmic drug delivery system.

## 1. Introduction

Various immune disorders are caused by immunopathological changes in the eye, collectively known as immune eye diseases, which include corneal transplant rejection, uveitis, age-related macular degeneration, retinitis, diabetic retinopathy, and macular edema. The human eye comprises anatomical structures and physiological barriers that prevent the drug from reaching the pathological site (Madni et al., 2017). There are several administration methods, such as local drip application and local injection (anterior chamber, subconjunctival, and vitreous). As injections are risky, drips in the anterior segment are the most accepted by patients, especially for chronic eye diseases that require long-term treatment. Drip administration could also utilize a non-corneal route (sclera) for retroocular delivery. Therefore, molecules’ water solubility determines their transport through the sclera and their ability to escape conjunctival blood and lymphatic washout (Aydemir et al., 2004).

Immunosuppressants are a large class of chemical drugs that inhibit abnormal immune responses of the body and the proliferation of immune response cells (macrophages, such as T cells and B cells) by suppressing antibody production (Liu et al., 2016). In organ transplantation, postoperative immune rejection can inhibit the expansion and proliferation of cytokines. Inhibiting autoimmune responses can happen at many levels, including cell subsets, cytokines, and immune cells (Wang et al., 2021). It has been shown that immunosuppressive drugs can reduce rejection of transplanted organs, increase graft survival rates, and extend the life span of recipients (Bauer et al., 2020). When combined with antibiotics, it has the ability to effectively inhibit inflammation. Furthermore, immunosuppressive agents are effective in the treatment of cancer (Rollan et al., 2022) and some diseases caused by abnormal autoimmunity (Jayatilleke, 2022). Comparatively to other therapies, immunosuppressive therapies target immune cells, specifically regulating their immune response. Some researchers have studied the disorders of neuromyelitis optica pedigree during pregnancy. According to the findings, women without immunotherapy were more likely to recur the disease in the early postpartum period, and postpartum disability was more severe (Shi et al., 2017). Immunosuppressive therapy during pregnancy and postpartum can reduce the risk of recurrence and the degree of disability. Besides, researchers have used immunosuppressive therapy to treat patients with aplastic anemia secondary to cancer chemotherapy or radiotherapy, and 50% of patients have alleviated their aplastic anemia (Nakagawa et al., 2021). The overall survival rate was significantly different from that of patients without immunosuppressive therapy (*p* < 0.05).

Immune rejection caused by organ transplantation (including corneal transplantation) is most commonly treated through systemic administration, such as oral administration, which is prone to causing systemic immunosuppression and serious adverse reactions. The immunosuppressants may show organ specificity and/or systemic toxicity, such as hepatorenal toxicity, hypertension, new endpoint diabetes after transplantation (NODAT) or post-transplant diabetes media (PTDM) (Davidson et al., 2003), dyslipidemia (Laish et al., 2011, Lucey et al., 2013), increased risk of cardiovascular disease (Wojciechowski & Wiseman, 2021). Additionally, they also have been implicated in the pathogenesis of diabetes, hypertension, hyperlipidemia, and cosmetic stigma. Immunosuppressants mainly include corticosteroids, alkylating agents, antimetabolic agents, calcineurin inhibitors and mammalian target of rapamycin (mTOR) inhibitors. Table 1 listed the classification of immunosuppressants, representative drugs and typical adverse reactions. Antibiotics are also required in organ transplantation to prevent infection, which may weaken immunity. Antimetabolic drugs are mainly used in severe patients, which can directly kill immune cells. They have a great inhibitory effect, accompanied by a great number of side effects, such as gastrointestinal superset, cytopenia, and hepatitis. Alkylating agents increase the risk of cancer related mortality. Therefore, it is recommended that the duration of alkylating agent treatment should not exceed 18 to 24 months (Guillevin et al., 1997). Besides, the side effects of corticosteroids are mainly manifested in the development of obesity, glucose intolerance, osteoporosis, ischemic necrosis, linear growth disorder (children), glaucoma, cataract, myopathy, and neuropsychiatric complications after transplantation (Danovitch, 2005). The acute toxicity of calcineurin inhibitors includes hypertension, renal dysfunction, and neurological disorders such as tremor and seizures (Bennett et al., 1996). Moreover, they play a role in the development of diabetes, hypertension, hyperlipidemia (Nankivell et al., 2003). mTOR inhibitors can lead to hyperglycemia, dyslipidemia, neurotoxicity, stomatitis, poor wound healing, pneumonia, vascular edema, lymphedema, Osteonecrosis (Nguyen et al., 2019).

**Table 1.** Classification, representative drugs and typical side effects of immunosuppressive drugs.

Class	Generic Name	Adverse Effects
Anti-metabolism	Azathioprine Methotrexate	Gastrointestinal upset, cytopenia, hepatitis.
Alkylate	Chlorambucil Cyclophosphamide	An increased risk of cancer related mortality.
Corticosteroid	Prednisone, Cortisone	Osteoporosis, ischemic necrosis, glaucoma, cataract, myopathy.
Calmodulin inhibitor	Cyclosporine Tacrolimus	Hypertension, renal dysfunction, neurological disorders
mTOR inhibitor	Sirolimus (rapamycin) Everolimus	Hyperglycemia, poor wind hearing nephrotoxicity, bone necrosis.

Although these immunosuppressants cause many side effects, some drugs are not recommended for clinical use. For examples, antimetabolic drugs cause too much harm to the human body. It is rare to use antimetabolic drugs as part of immunosuppressive therapy. The risk of alkylating agents to cancer has led people to advocate non alkylating agents (Jabs, 2018). In addition, the main risks of corticosteroid use in the eyes are cataract, infection tendency and elevated intraocular pressure, which may lead to glaucoma (Burkholder & Jabs, 2021). As compared to the first three, this calcineurin inhibitor and mTOR inhibitor have relatively few side effects, including hepatotoxicity, hypertension, bone necrosis and other systemic side effects. The preparation of local eye nano preparation can better increase the eye targeting and reduce the side effects on the whole body. Therefore, clinical treatment drugs for ocular immune rejection are being developed in a hotspot (Cotovio et al., 2012).

**Figure 1.**
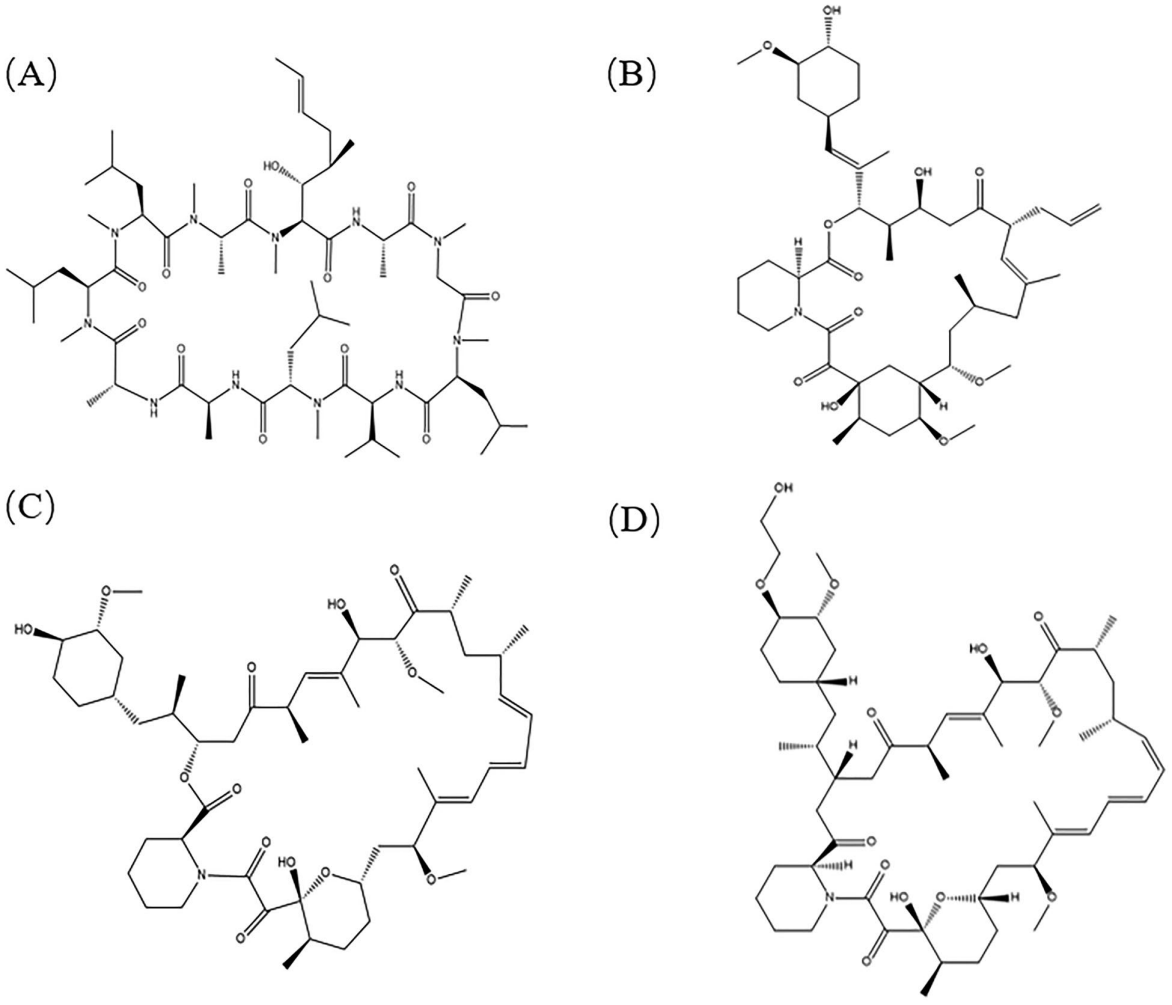
Structural formulas of cyclosporine, tacrolimus, sirolimus, and everolimus. (A) Cyclosporine, molecular weight 1202.61, solubility <10 μg/mL); (B) Tacrolimus (molecular weight 804.02, solubility 5-8 μg/mL); (C) Sirolimus (molecular weight 914.172, solubility 2.6 μg/mL); and (D) Everolimus (molecular weight 958.2, solubility 9.6 μg/mL).

The most commonly used ocular immunosuppressants are cyclosporine (cyclosporine A, CsA), tacrolimus, sirolimus (rapamycin), and everolimus. In addition to corneal transplant rejection, these immunosuppressants can also be used to treat scleritis, macular degeneration, uveitis, and dry ophthalmopathy. The blood-eye barrier limits the ocular bioavailability of systemic medications, whereas all four immunosuppressants are highly lipophilic and low in water solubility when administered as topical eye drops (solubility in water < 10 μg/mL, large molecular weight, approximately 1000 g/mol). Figure 1 depicts the structural formulas of some ocular immunosuppressants. Therefore, drugs prepared in various formulations, such as nanosuspension, nanoparticles, liposomes, micelles, and implants, can improve their solubility in water, reduce their toxicity, and facilitate their delivery.

**Figure 2.**
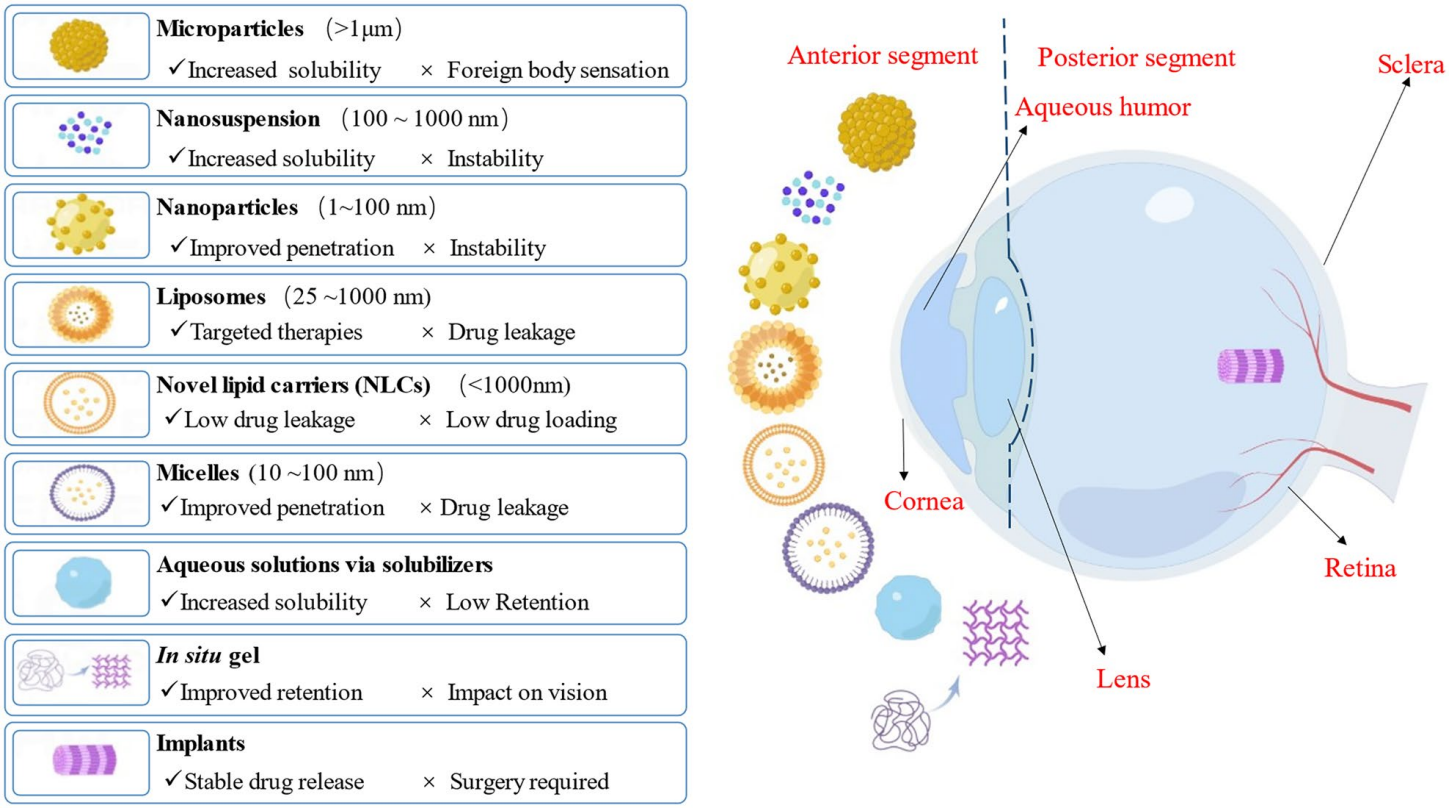
Schematic representation of novel ophthalmic dosage forms. By Figdraw, ID:YUUSAa7ea7.

The particle sizes range varies considerably from formulation to formulation, as shown in Figure 2. Microparticles include microspheres and microcapsules with particle sizes measured in micrometers. Microspheres are spherical particles formed by dispersing drugs in polymer materials, whereas microcapsules are microscopic capsules created by encapsulating drugs in polymer capsules. Notably, if the particle size of the particles administered to the eye exceeds 10 μm, a foreign body sensation will occur. Nanosuspensions are nano-controlled release systems consisting of colloidal discrete systems stabilized by surfactant (Wang et al., 2016). A nanosuspension protects solutions with poor dissolvability in lachrymal fluids with particle sizes between 100 nm and 1000 nm. Compared to conventional ocular formulations, nanosuspensions have reduced particle sizes and increased specific surface areas. Typically, nanoparticles have a radius or characteristic size below 100 nm. Hybrid nanoparticles may penetrate posterior ocular tissues more effectively because of their hydrophilic properties. Liposomes are microscopic vesicles composed of phospholipid bilayers that enclose aqueous compartments with particle sizes from 25 nm to 1000 nm. As liposomes can be continuously administered and form deposits within the cornea, they have been widely used for targeted therapies for a long time (Karn et al., 2014). Unfortunately, some studies have mentioned that because of liposomes’ short half-life at the corneal surface, the concentrations of CsA in aqueous and vitreous humor after liposome treatment were low, leading to the release of free CsA before liposome penetration into the cornea (Nikoofal-Sahlabadi et al., 2013). Therefore, CsA cannot enter the posterior end of the eye for treatment. Novel lipid carriers (NLCs) are formulated from a mixture of solid and liquid lipids, which prevents drug leakage. NLCs have good biocompatibility and can be produced on a large scale through high-pressure homogenization compared with other colloidal carriers (Li et al., 2008); however, the addition of many excipients resulted in low drug loading efficiency. Besides NLCs, polymeric micelles can be used to improve corneal and conjunctival penetration, maintain drug levels, and reduce systemic side effects. Micelles are amphiphilic spheres self-assembled by surfactants with a radius from 10 nm to 100 nm (Kwon, 2003). The surfactants create a hydrophobic core that encloses the hydrophobic molecules. Micelles are more dynamic than polymeric nanoparticles. Nevertheless, drug leakage may occur during storage and transportation because of the drug diffusing out of micelles as the temperature fluctuates (Crean 2009). In some studies, insoluble drugs were prepared as aqueous solutions via solubilizers through a straightforward process. However, none of the aforementioned preparations can remain in the eye for a long time because of the eye’s unique structure; most of the drugs are lost on the ocular surface and are unable to exert better efficacy. *In situ* gel is a kind of preparation that undergoes phase transition immediately at the application site after administration in the solution state, forming a non-chemically cross-linked semi-solid gel from the liquid state. *In vitro* gel—the substance is in a liquid state—facilitates canning and transport. It is converted into a gel at the application site, thereby increasing the retention time in the eye, reducing administration frequency, and improving patient compliance. However, some *in situ* gels can impair vision. In addition to these nanomedicines, eye implants comprise a mixture of drugs and polymeric materials contained in a miniature device or prepared according to a certain formulation. With accurate dosing, stable drug release, and high targeting, implants are surgically transplanted into the eye. A small drug dose could produce therapeutic effects. If the implant causes an adverse reaction or is no longer needed, it can be removed. Biodegradable implants can be degraded without surgical removal.

**Figure 3.**
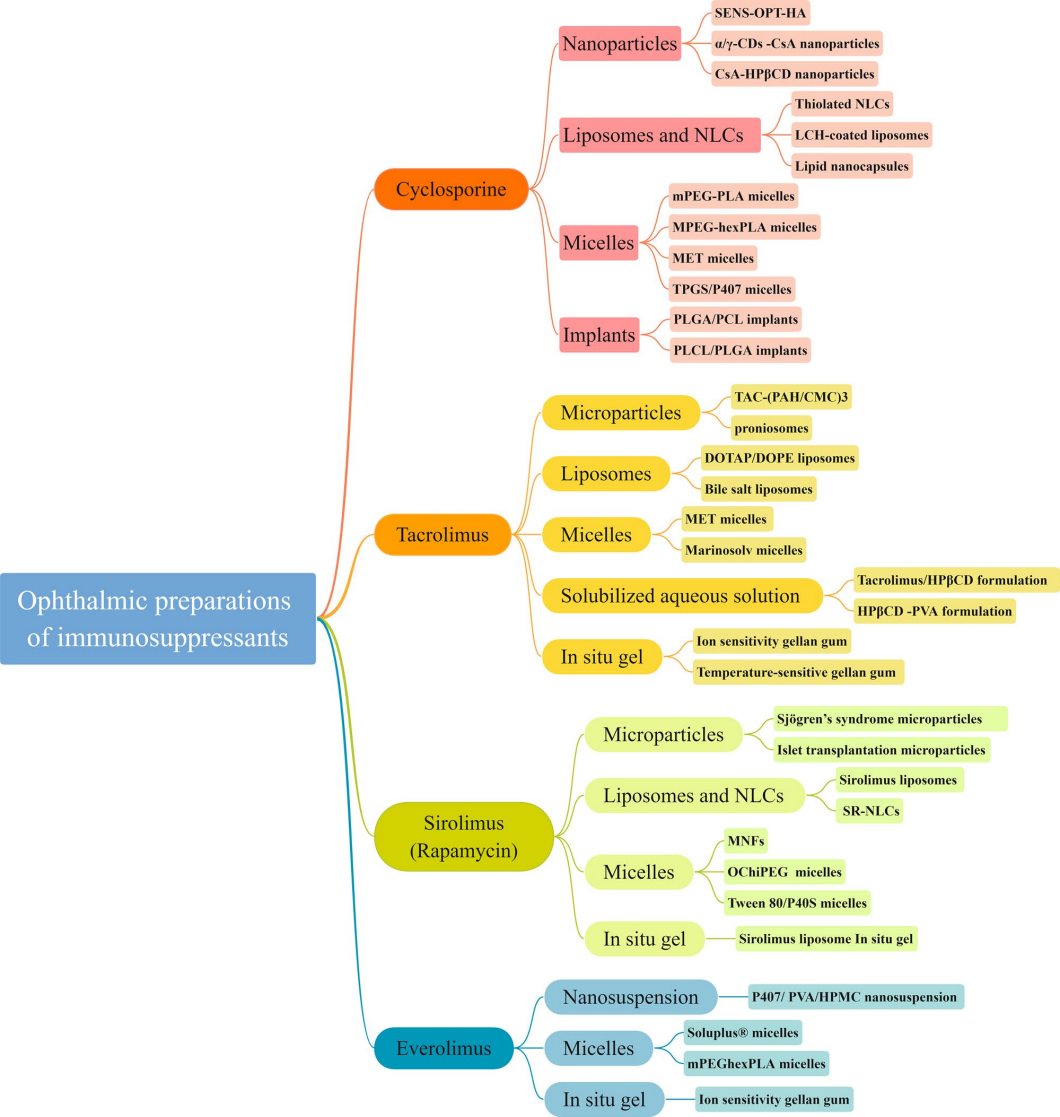
Immunosuppressant types and formulations.

This article reviews the ophthalmic preparations of these four drugs as a reference for future research and development just as Figure 3 shown.

## 2. Cyclosporine

Cyclosporine (CsA), also known as cyclosporine polypeptide, is an immunosuppressant that interferes with T-helper cell activation by blocking interleukin-2 production. When CsA enters T cells, it can bind to the cyclophilin cytoplasmic protein receptor, forming a cyclosporine-cyclophilin complex. IL-2 gene expression, Ca^2+^-dependent serine/threonine phosphokinase activity, and T cell activation are inhibited through the inhibition of calcineurin-phosphatase activity (Jones et al., 2017, Pflugfelder & De Paiva, 2017). CsA can be used to treat various ocular immune diseases, including necrotizing scleritis, Behçet’s syndrome, severe vernal keratoconjunctivitis, conjunctivitis, dry eye syndrome, and treatment after high-risk corneal transplantation. In contrast to corticosteroids, CsA is safe for long-term use without the negative effects associated with other anti-inflammatory drugs (Utine et al., 2010). CsA treatment is commonly associated with a burning sensation in the eye, blurred vision, eye itch, conjunctival hyperemia, discharge, tingling, and foreign body sensations (Palestine et al., 1984). Conventionally, CsA has been administered orally for treating ophthalmologic diseases. However, topical administration is now preferred because of the systemic side effects. In addition to reducing systemic adverse effects, it also improves bioavailability and specific targeting of ocular tissues (Sahoo et al., 2008). CsA is very poorly soluble in water at physiological temperatures (only 4 μg/mL), whereas its solubility is improved at refrigeration temperatures (134 μg/mL). Therefore, it is primarily necessary to increase solubility, trans-corneal penetration, and precorneal residence time, while reducing dosing frequency.

The first CsA formulation currently available for treating human dry eye syndrome is the FDA-approved topical emulsion (Restasis®, Allergan). Glycerin and castor oil are used to improve the aqueous solubility and corneal permeability of CsA. However, the additives often cause side effects such as visual disturbances, conjunctival hyperemia, and ocular burning (Mccoy, 2015). In some cases, these additives can cause high irritation because they tend to affect the integrality of the ocular tissues, limiting their further clinical application. Ikervis®, a cationic ophthalmic emulsion containing 0.1% CsA, was approved by the European Commission in 2015 for treating severe keratitis in adults with dry eye syndrome that could not be improved with artificial tears. It comprises medium-chain triglycerides, cefaclor, glycerin, tyloxapols, and poloxamer 188. It primarily works through the special electrostatic interaction between negatively charged corneal epithelium and ocular drugs (Daull et al., 2014). However, the drug caused some side effects, such as burning and stinging eyes, which occurred in 5.9% of patients in blinded clinical trials (European Medicines Agency, 2015). One study (Daull et al., 2016) divided dry-eye mice into four groups: one control group and three experimental groups. Each experiment group received either 1% methylprednisolone (glucocorticoid), 1% Ikervis® emulsion, or 1% Ikervis® vehicle. Ikervis® significantly improved tear volume by more than 1% methylprednisolone or Ikervis® vehicle. Corneal fluorescein staining was used to check for corneal epithelial defects. A lower score indicated less severe corneal damage. Compared with the control group, the three experimental groups demonstrated an average decrease in corneal fluorescein staining.

### 2.1. Nanoparticles

Hyaluronic acid (HA) is a nonirritating, highly water-binding, viscous, and pseudoplastic substance (Contreras-Ruiz et al., 2011). It may enhance ocular retention with mucoadhesive properties, thereby increasing the effectiveness of the drug through the cornea. HA coatings enhance nanoparticle uptake of corneal cells through their specific targeting of HA receptors. Span80, tocopherols polyethylene glycol 1000 succinate (TPGS), and cetyltrimethylammonium-ammonium bromide (CTAB) were found to dissolve in absolute ethanol (Alvarez-Trabado et al., 2018). The ethanol was poured steadily over purified water under continuous magnetic stirring, resulting in the spontaneous formation of Sorbian ester nanoparticles (SENS). Box-Behnken experimental design was enforced to obtain a cationic-optimized formulation (SENS-OPT). Moreover, the SENS-OPT was coated with HA to achieve anionic optimization (SENS-OPT-HA). The SENS-OPT was 170.5 nm in diameter, with a Zeta potential of +33.9 mV and a CsA loading of 19.66%. Because of the HA coating, the Zeta potential was −20.6 mV, and the particle size was 177.6 nm. The SENS-OPT-HA induced greater cellular uptake and greater penetration in human corneal epithelial (HCE) cells across porcine corneal tissues compared to SENS-OPT without coating. In biocompatibility studies, both two formulations were found to be safe.

In addition to HA, cyclic oligosaccharide cyclodextrins (CDs) are also a type of common material used in nanoparticles. CDs have been reported to improve the physicochemical and biopharmaceutical properties of peptides and proteins. CDs are ideal for forming inclusion complexes with lipophilic drugs because of their lipophilic cavity and hydrophilic exterior, thereby increasing their water solubility. CDs can enhance drug permeability through corneal membranes by permeating the membrane or combining it with lipophilic components such as cholesterol and phospholipids (Shimpi et al., 2005). As the α-CD in natural foods can solubilize CsA at 0.5 mg/mL and promote the formation of nanosized CsA/CD particles (Jóhannsdóttir et al., 2015). In addition to α-CD, γ-CD is a CD with higher water solubility and a better toxicological profile than other pharmaceutically acceptable CDs, making it an attractive solubilizer for ophthalmic preparations. Researchers have prepared CsA nanoparticles (0.2% w/v) mixed with α-CD and γ-CD. During a 3-month period, the eye drops were administered to rabbits once or twice per day without any toxic side effects or ocular irritation (Jóhannsdóttir et al., 2017). Moreover, 2-hydroxypropyl-β-cyclodextrin (HPβCD) is a cyclic oligosaccharide comprising seven units of α-1,4-linked glucose and a hydroxy propylated group, with a lipophilic core and a hydrophilic exterior (Loftsson et al., 1989). The European Medicines Agency (EMA) selected HPβCD as an ophthalmic excipient because HPβCD is the safest and most appropriate form of cyclodextrin for topical use with no toxicity (European Medicines Agency, 2017). Research has demonstrated that the CsA-HPβCD complex was prepared by kneading and triturating. The CsA-HPβCD complex (10 mg) and poly-lactide-co-glycoside (PLGA) were then dissolved in dichloromethane. With an ultrasound probe, this mixture was first emulsified in purified water containing 1% (w/v) polyvinyl alcohol (PVA) and then poured into a polyvinyl alcohol aqueous solution. The dichloromethane was removed by stirring. To resuspend the nanoparticles, the evaporating nanoparticles were centrifuged at 14000 rpm and resuspended in mannitol solution after evaporation. The CsA encapsulated nanoparticles had a high encapsulation efficiency (88%), a high yield (89%), and a high release rate (75–96%) over 24 hours (Aksungur et al., 2012).

### 2.2. Liposomes and NLCs

According to a study, NLCs with thiolated modifications could prolong corneal retention time *in vivo*, whereas the thiolated NLCs would not induce discomfort or irritation and would deliver a sustained release of CsA *in vitro* (Shen et al., 2010). *In vivo* distribution studies have demonstrated that CsA concentration in the systemic blood was very low and almost undetectable. Compared to oil solution, non-thiolated NLCs, the area-under-the-curve (AUC_0–24 h_) and mean retention time (MRT_0–24 h_) of CsA-NLCs in aqueous humor, tears, and eye tissues were significantly higher. Thiolated NLCs could deliver high levels of CsA into the ocular surface and anterior chamber tissues and prolong pre-corneal residence time because of their sustained release characteristics and bio-adhesive property.

As a chitosan derivate, low molecular weight chitosan (LCH) exhibits a remarkable improvement in water-solubility compared to its precursor (Li et al., 2005). It was proposed that LCH-coated liposomes (LCHL) could deliver drugs to the ocular cavity with an injection method. Based on dynamic light scattering instrument measurements of LCHL applied to CsA, the average particle size was 89.6 ± 2.3 nm. Rabbit corneal epithelial cells exhibited low toxicity to LCHL. A delayed drug release profile for LCHL was measured *in vitro*. LCHL significantly increased CsA concentrations in the cornea, conjunctiva, and sclera of rabbits *in vivo* (Li et al., 2012).

Lipid nano capsules (LNCs), one category of NLCs, consist of a lipid core surrounded by a rigid membrane containing lecithin and pegylated surfactant (Huynh et al., 2009). By combining polymeric nano capsules and liposomes, a hybrid structure can be created. In addition to their good colloidal properties, LNCs exhibit a narrow dispersity range and a size range of 20–100 nm. As most aqueous ophthalmic preparations are washed out with tears, a thermosensitive gel containing CsA encapsulated in LNCs was developed with an average CsA-LNC particle size of 41.9 ± 4.0 nm. The CsA-LNC was narrow in size distribution (PDI = 0.1) with a high entrapment efficiency (over 98%). CsA-LNC *in situ* gels were prepared using poloxamer 407 (P407). The Draize test was used to compare the effects of Restasis® and CsA in castor oil on ocular irritation in rabbits. Compared to the other formulations tested, LNC incorporation into *in situ* gels led to increased adhesiveness and stronger corneal fluorescence. According to Schirmer tear test results, CsA-LNC formulations recovered more quickly compared to Restasis®. The visual and histopathological examinations of CsA-LNC revealed no ocular irritation (Eldesouky et al., 2021).

### 2.3. Micelles

There are three key excipients used to prepare micelles.

1. Methoxy poly (ethylene glycol)-poly (lactide) polymer (mPEG-PLA), a biodegradable polymer approved by FDA, has been used in clinical trials for micelles (Panahi et al., 2017). The research reports have indicated that mPEG-PLA can reduce protein absorption, making it suitable for nano colloid formulations for ophthalmic drugs (Giannavola et al., 2003). Micelles coated with mPEG-PLA were found to exhibit minimal cytotoxicity, non-irritation to the eye, and increased drug penetration (Phan et al., 2016). As a steric stabilizer, mPEG2000 mainly protects micelles during lyophilization. By adding mPEG2000 to the micellar solution, the gaps between micelles and micellar solutions can be filled with polyethylene glycol chains. The lyophilized micelles containing 5% mPEG2000 maintained chemical and physical stability when stored at 4 °C for 3 months. Meanwhile, mPEG-PLA micelles were released *in vitro* in a delayed and sustained manner. A cytotoxicity study proved that the mPEG-PLA blank micelles were nontoxic. Cellular uptake demonstrated that energy played a role in clathrin-mediated endocytosis. In the rabbit corneal, CsA-loaded mPEG-PLA micelles had a maximum concentration of 4.5-fold higher than that of Restasis® administration and, after 8 hours, still 0.8% CsA remained stranded. mPEG-PLA micelles exhibited a better retention effect in ocular distribution studies and *in vivo* pharmacokinetic profiles (Yu et al., 2018).
2. The methoxy poly (ethylene glycol)-poly (hexyl-lactide) (MPEG-hexPLA) micelles (Di Tommaso et al., 2011) were prepared using the co-solvent method. MPEG-hexPLA copolymer dissolved in acetone was added dropwise to PBS containing 10% sucrose under sonication. The acetone evaporation at room temperature yielded a micelle solution containing a copolymer (10 mg/mL). The cytotoxicity assay and clonogenic test demonstrated that MPEG-hexPLA micelles were biocompatible *in vitro* and *in vivo*, as well as nontoxic on HCE cells. Further, immunohistochemistry analysis revealed no activation of apoptosis mechanisms. MPEG-hexPLA micelles have good ocular biocompatibility, suggesting that they can be used for the eyes.
3. MET polymers, N-palmitoyl-N-monomethyl-N, N-dimethyl-N, and N, N-trimethyl-6-O-glycolchitosan (Schtzlein, 2021) can encapsulate CsA in aqueous phases through high-pressure homogenization because they are viscose self-assembling micelle materials that contain hydrophobic groups along their side. During storage at refrigeration temperatures and room temperatures, micelles containing 0.01%, 0.02%, and 0.05% CsA were stable for 28 days. Because of topical application with MET micelles containing 0.05% CsA to rabbits, AUC_0–24h_ levels were 25780, 12046, and 5879 ng·h/g in the corneal, conjunctival and scleral membranes, respectively. Similarly, Restasis ® with 0.05% CsA produced lower levels of 4726, 4813, and 1729 ng·h/g, respectively, in the corneal, conjunctival, and scleral membranes of the rabbits. The results demonstrated that MET micelles were more effective than commercial formulations in terms of increasing drug levels.
4. TPGS and poloxamer p407 were used to prepare mixed micelles at a ratio of 1:1, which had sufficient rheological properties and good eye compatibility at 35 °C (Grimaudo et al., 2018). By using 20 mM p407, the apparent solubility of cyclosporine was increased 107-fold (higher than the CMC of mixed micelles). The concentration of cyclosporine was 6.4 ± 1.9, 17.6 ± 5.4 and 26.9 ± 7.4 µ g/g cornea respectively with the mixed micelles of 1, 2.5 and 4 mg/ml cyclosporine concentration penetrated the isolated porcine cornea for 3 hours. The micelles containing 4 mg/ml cyclosporine were used for the penetration of sclera in vitro. Cyclosporine accumulated 28 ± 7, 38 ± 10, 57 ± 9, 145 ± 27 µg/cm^2^ after 3, 6, 24, and 48 hours, respectively.

### 2.4. Implants

Sustained-release CsA formulations for subconjunctival delivery were developed by loading PLGA and PCL nanoparticles onto implants (Pehlivan et al., 2015). CsA-loaded nanoparticles were loaded into implants to prolong their release time, and CsA levels may be therapeutically achievable using this system in approximately 15 days (Apel et al., 1995). For manufacturing PLGA implants loaded with CsA, a copolymer (lactide: glycolide = 85:15) was used with the electrospinning process, which is mainly used for the production of nano and microfibers of polymeric materials. Fiber structure and morphology can be controlled by modifying several parameters, such as polymer type and solution concentration, because of this method. A two-nozzle electrospinning method was used to load PLGA-CsA nanoparticles into PCL fiber structures. The sustained-release implants were able to deliver CsA for up to 90 days. Efficacy studies have also demonstrated that implants and fibers containing CsA helped to accelerate the healing process.

**Figure 4.**
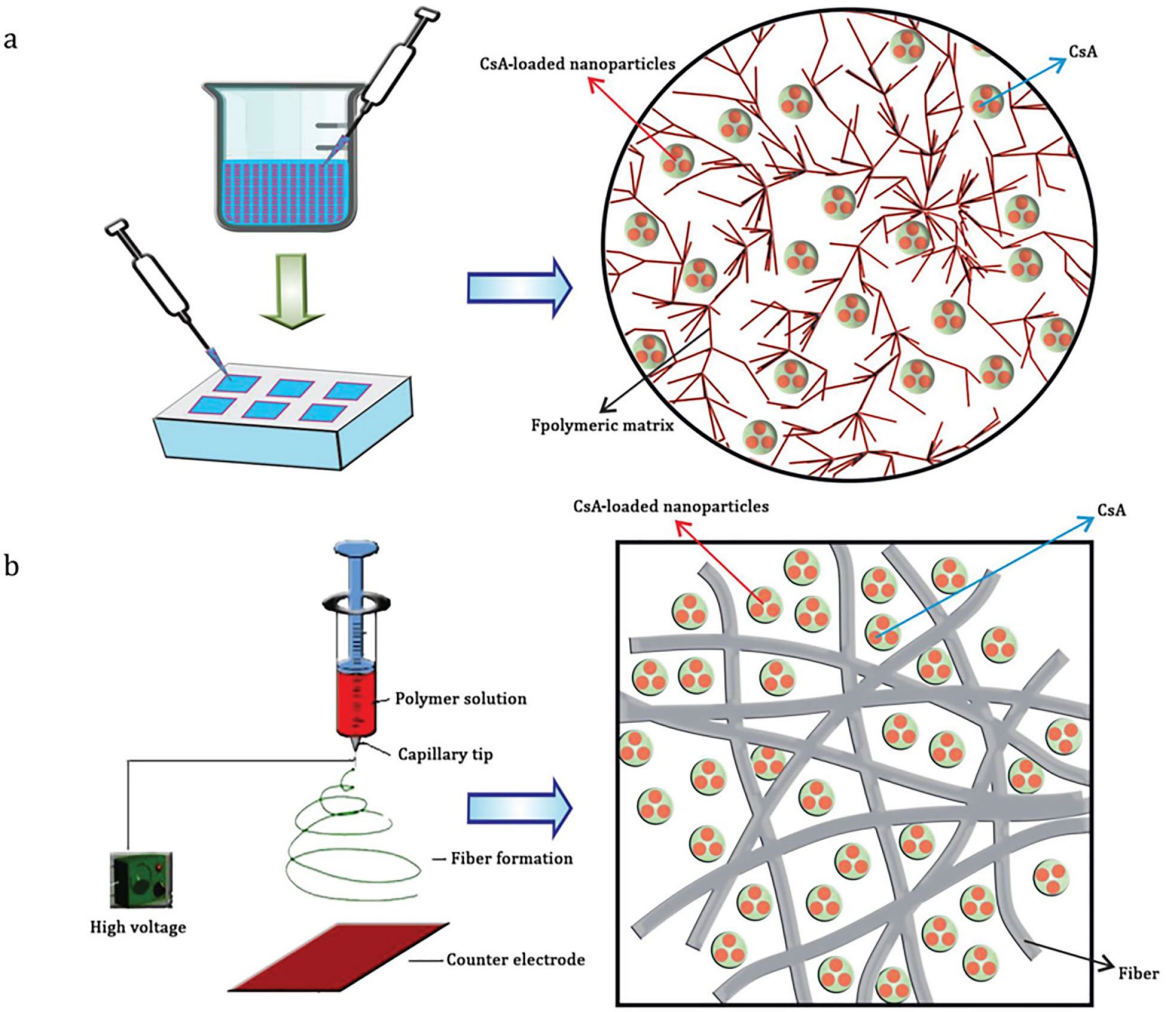
Schematic diagram of implant preparation: (a) molding method and (b) electrospinning method (Yavuz et al., 2016).Copyright 2016, Drug Delivery.

Furthermore, scholars have mentioned that the molding method was one of the most commonly used implant preparation techniques and that it was compared with the electrospinning method (Yavuz et al., 2016). Figure 4 illustrates two methods of implant preparation. This technique was most commonly used for fiber preparation for tissue engineering. Molding and electrospinning were used to prepare poly (lactide-co-caprolactone) (PLCL) and PLGA nanoparticle-loaded implants. The *in vitro* characterization results revealed that both techniques were effective at loading CsA into nanoparticle-loaded implant formulations. *In vitro* studies have shown that CsA can be released in PBS at 37 °C for two months. Scanning electron microscope imaging revealed a more uniform distribution of nanoparticles in the fiber implants. However, *in vitro* degradation results and morphological structures indicated that mold-based implant formulations release significantly longer than fiber-based formulations. Molded implants were chosen for preliminary efficacy testing in Swiss Albino mice with induced dry eye syndrome because of their longer release duration. Compared with the control group (blank implants), molded implants accelerated healing over a 90-day evaluation period.

## 3. Tacrolimus

Tacrolimus, also known as FK506, is a high-molecular-weight (804.02 g/mol) hydrophobic macrolide immunosuppressant isolated from *Streptomyces tsukubaensis*. As a 23-membered macrolide antibiotic, it is a powerful immunosuppressant with good tissue penetration when administered locally to the eye. It acts similarly to CsA, but it is 10–100 times stronger than CsA. They both inhibit calcineurin and prevent the activation of T cells. By inhibiting the release of inflammatory cytokines, tacrolimus suppresses the immune system by inhibiting histamine release from mast cells (Vichyanond & Kosrirukvongs, 2013). It is often used to treat severe refractory uveitis, allograft rejection, dry eye syndrome, and vernal keratoconjunctivitis. According to previous studies, tacrolimus is effective in improving eye disease without causing any adverse effects. It is also less likely to cause hypertension and dyslipidemia than CsA. Steroids can inhibit corneal allograft rejection, but morbidity rates remain high, with complications such as systemic infections, cataracts, and glaucoma. Patients who are resistant to steroids and CsA can benefit from tacrolimus. Nevertheless, tacrolimus has some difficulty in reaching effective therapeutic intraocular concentrations and penetrating the cornea because of its highly hydrophobic character(5–8 μg/mL)and high molecular weight (804.02). Moreover, systemic administration of tacrolimus may cause severe side effects, such as hyperglycemia, nephrotoxicity, neurotoxicity, weight loss, liver damage, and diarrhea (Shapiro et al., 1990). Therefore, its preparation as an ophthalmic preparation is urgent.

Talymus® is the commercially available ophthalmic formulation of tacrolimus for treating vernal keratoconjunctivitis. Manufactured by Japan’s Senju Pharmaceutical Co., Ltd., Talymus® was approved for marketing in Japan in 2008 and for import in China in June 2013. The primary excipient is polyvinyl alcohol (partially saponified), which acts as a suspension aid. This product also contains other excipients, including preservatives (benzalkonium chloride), osmotic pressure regulators, and pH regulators (sodium chloride, sodium hydrogen phosphate hydrate, sodium phosphate, and phosphate). Tacrolimus is dispersed in the formulation in fine particles, whose particle size has an impact on the bioavailability and comfort of the medicine. Besides, 0.03% Tacrolimus eye drops used by researchers as a control in the experiment were obtained by reformulating intravenous drug presentation (Prograf®) with ethanol, containing irritating compounds that are typically unpleasant for the patient (Luaces-Rodríguez et al., 2018).

### 3.1. Microparticles

As a lubricant and viscosity-enhancing agent in artificial tears, carboxymethyl cellulose (CMC) is one of the most commonly used cellulose derivatives with carboxymethyl groups (Coursey et al., 2015). CMC can attach itself to corneal epithelial cells and increase the residence time on the cornea because of its mucoadhesive property. Poly (allylamine hydrochloride) (PAH) was alternately deposited to prepare the CMC coating. 1% PVA could confer well-dispersed tacrolimus microcrystals (TAC-MCs) with hexagonal plate-like shapes. Nonspherical TAC-MCs were synthesized by precipitation method and layer-by-layer coated with CMC to produce TAC- (PAH/CMC)_3_. As a rule of thumb, TAC-MCs measure 8.90 ± 0.35 µm in length and 3.89 ± 0.19 µm in width. TAC- (PAH/CMC)_3_, followed by TAC MCs, had the higher therapeutic efficiency than the commercial eye drops Talymus®, which includes improvement of preservation of tissue structure, clinical dry eye signs, and suppression of the expression of representative inflammatory mediators. TAC- (PAH/CMC)_3_ is notable for its superior therapeutic performance compared with Talymus®, which is attributed to the lubricant, mucoadhesive effect of CMC, the non-spherical geometry of MCs, and the anti-inflammatory function of tacrolimus (Zhang et al., 2020).

Niosomes are nonionic surfactant and lipid structures that can accommodate hydrophilic and hydrophobic drugs (Abdelbary & El-Gendy 2008). Aside from being more cost-efficient, they also offer lower toxicity and easier formulation without the use of unacceptable solvents compared with other vesicular drug delivery systems. However, niosomes exhibit physical and chemical instability during storage, including leakage or hydrolysis of encapsulated drugs, sedimentation, fusion, and aggregation, thereby affecting the shelf life. Generally, proniosomes can form a stable semisolid gel structure (Sankar et al., 2010), which prevents encapsulated drugs from hydrolyzing during storage and transportation (Ammar et al., 2011). Proniosomes were prepared as follows: an ethanol solution containing cholesterol, poloxamer 188, and lecithin in a ratio of 1:9:9 was mixed with tacrolimus at 65 °C for 10 minutes. After adding PBS to the tube, the solution was kept for about 5 minutes until it became clear. After cooling to room temperature, the solution formed a proniosomal gel. Dynamic light scattering measured the mean proniosome size as 1.33 ± 0.32 μm with a polydispersity index (PDI) of 0.21 ± 0.03. Proniosome-derived tacrolimus exhibited an entrapment efficiency of 95.34 ± 0.02%. The increase in tacrolimus permeation through freshly excised rabbit corneas and tacrolimus retention in corneas was observed. The instillation of 1% tacrolimus-containing proniosomes four times per day *in vivo* did not cause irritation or corneal damage in a 21-day *in vivo* test. Anti-allograft rejection in SD rats following corneal xenotransplantation was assessed *in vivo*. The results demonstrated that 1% tacrolimus-containing proniosomes delayed corneal allograft rejection and significantly prolonged overall corneal allograft survival to 13.86 ± 0.80 days compared with those treated with either 1% CsA eye drops, blank proniosomes, or those left untreated.

### 3.2. Liposomes

A mucin layer on the ocular surface makes the surface an anionic environment, which predisposes the surface to attract cationic liposomes electrostatically (Chen et al., 2022). (2,3-dioleoyloxy-propyl)-trimethylammonium (DOTAP) is one type of cationic phospholipid used to encapsulate tacrolimus. 1,2-Dioleoyl-sn-glycero-3-phosphoethanolamine (DOPE) is a neutral co-lipid for cationic liposomes. Tacrolimus liposomes had a positive charge on their surface of +30 mV and a diameter of approximately 300 nm. HCE cells with tacrolimus liposomes concentrations below 0.2 mg/mL exhibited good viability after co-incubation for 24 hours. For rabbits treated with 0.2 mg/mL of the drug, their corneal thickness and endothelial cell density remained unchanged for 28 days. No significant difference was observed between the PBS group and the tacrolimus liposome group for cell safety. Moreover, tacrolimus liposomes could reduce the expression of inflammatory factors associated with dry eyes of rabbits and C57BL/6 mice, as well as alleviate the signs of dry eye.

Researches have shown that using bile salts instead of cholesterol can make liposomes more flexible, easier for the carrier to penetrate biofilms (Niu et al., 2011). Compared with traditional liposomes, liposomes containing bile salts improved oral (Guan et al., 2011) and percutaneous absorption (Li et al., 2011) of drug molecules. Glycocholic acid, deoxycholate, and taurocholate were less irritating and were suitable for drug delivery. Tacrolimus liposomes prepared by membrane dispersion method were approximately 100 nm in size, and over 90% of tacrolimus was encapsulated. Liposomes containing bile salts can deliver three to four times more tacrolimus in isolated cornea transport than conventional liposomes. Studies on cytotoxicity and corneal tolerance in vivo showed that liposomes containing sodium taurocholate or sodium glycocholate were well tolerated, while those containing sodium deoxycholate were toxic to rabbit cornea (Dai et al., 2013) .

### 3.3. Micelles

The MET polymer mentioned in Section 2.3 (3) has been reported to encapsulate tacrolimus with a thin-film hydration method (Badr et al., 2021). The particle size of micelles was approximately 200 nm. The osmolality value was within the acceptable range for the eyes (327 ± 3.05 mOsm/kg). When MET micelles are refrigerated, they do not appear to degrade within 7 days, displaying a similar drug concentration at day 0 (0.99 ± 0.002 mg/mL) as compared to day 7 (1.0 ± 0.01 mg/mL), but different from day 30 (0.94 ± 0.049 mg/mL). MET micelles (25 µL, 0.1% w/v) successfully promoted tacrolimus permeation into ocular tissues following the instillation of MET micelles (25 µL, 0.1% w/v). After a single instillation of MET micelles (25 µL, 0.1% w/v), MET micelles could promote the permeation of tacrolimus into ocular tissues. The drug was delivered to the cornea (4452 ± 2289 ng/g tissue) and conjunctiva (516 ± 180 ng/g tissue) at much higher concentrations than its therapeutic level (5–10 ng/mL). Additionally, MET micelles were administered ocularly at the same dose of 25 μL (0.1% w/v) without causing erythema, tearing, or swelling.

A novel intellectual property-protected technology platform, called Marinosolv (Siegl et al., 2019), can be used to dissolve lipophilic, nearly water-insoluble drugs such as tacrolimus and corticosteroids in a buffered aqueous solution (pH 6.0) in the form of micelles by adding saponins, such as glycyrrhizin from licorice root and escin from horse chestnut, and the additional use up to 10% of solvents, such as dexpanthenol and propylene glycol. With Marinosolv, tacrolimus can be dissolved in aqueous formulations up to 900 g/mL, resulting in a 300-fold increase in tacrolimus solubility (Nakowitsch et al., 2016). The formulation can be stored for at least 4 weeks at room temperature and several months at 2–8 °C. When tacrolimus dispersed in Marinosolv was injected into porcine eyes, tacrolimus concentration in the cornea was 10-fold higher than that of Talymus®.

### 3.4. Solubilized aqueous solution

Based on CD intrinsic properties, tacrolimus solubility was improved by approximately 42 times (Mahmoudi et al., 2020). The tacrolimus/HPβCD formulations were prepared by dissolving HPβCD and then adding tacrolimus (García-Otero et al., 2021). Nuclear Magnetic Resonance (NMR) and molecular modeling studies have reported that 0.02% tacrolimus concentration could be obtained by using 40% HPβCD aqueous solutions. Results of *ex vivo* bio adhesion revealed good mucoadhesive properties. Positron emission tomography/computed tomography imaging (PET/CT imaging) was used as a molecular imaging technique to investigate the *in vivo* formulations’ permanence on the corneal surface. According to the PET/CT studies, the formulations were mucoadhesive and maintained a long-term presence on the ocular surface owing to an adequate consistency. The time of *in vivo* ocular permanence was higher (t_1/2_ of 86.2 min for the 40% HPβCD eyedrop and t_1/2_ of 46.3 min for the reference formulation of 0.03% tacrolimus ointment Prograf®). This novel eye drop formulation offers high potential as a safe alternative and a versatile vehicle to incorporate new topical ophthalmic drugs.

Another tacrolimus/HP-βCD formulation (containing HP-βCD, tacrolimus, and PVA) was mixed in PBS and rotated for 24 hours at room temperature. After centrifuging and filtering the supernatant, the final solution was prepared. Over the course of 28 days, each eye received one drop of tacrolimus-prepared formulation and some placebo formulation every 12 hours. Clinical examinations on days 1, 3, 7, 14, and 28 of the study revealed conjunctivitis and transient redness. No statistically significant difference was observed between the two groups in terms of corneal epithelial defects, redness, or pathological evaluations (Mahmoudi et al., 2020).

### 3.5. *In situ* gel

Aluminum chloride (ALC) was used as a cross-linking agent to develop gellan gum based on ion sensitivity (Modi et al., 2022). The tacrolimus-gellan gum was efficiently encapsulated (74.2 ± 2.4%) with nano size (274.46 ± 8.90 nm) and a high loading capacity (36.14 ± 1.7%). According to Fourier transform infrared spectroscopy (FTIR), gellan gum did not interact with the drug. A histopathological evaluation, a HET-CAM study, and a Draize test indicated that the formulation was safe for ocular use. A prolonged drug release occurred from the tacrolimus-gellan gum throughout a 12-hour period. Higher pre-corneal retention was observed compared with tacrolimus solution dissolved in mineral oil. *In vivo* studies on rabbits demonstrated prolonged precorneal retention and enhanced noncorneal and corneal penetration.

**Figure 5.**
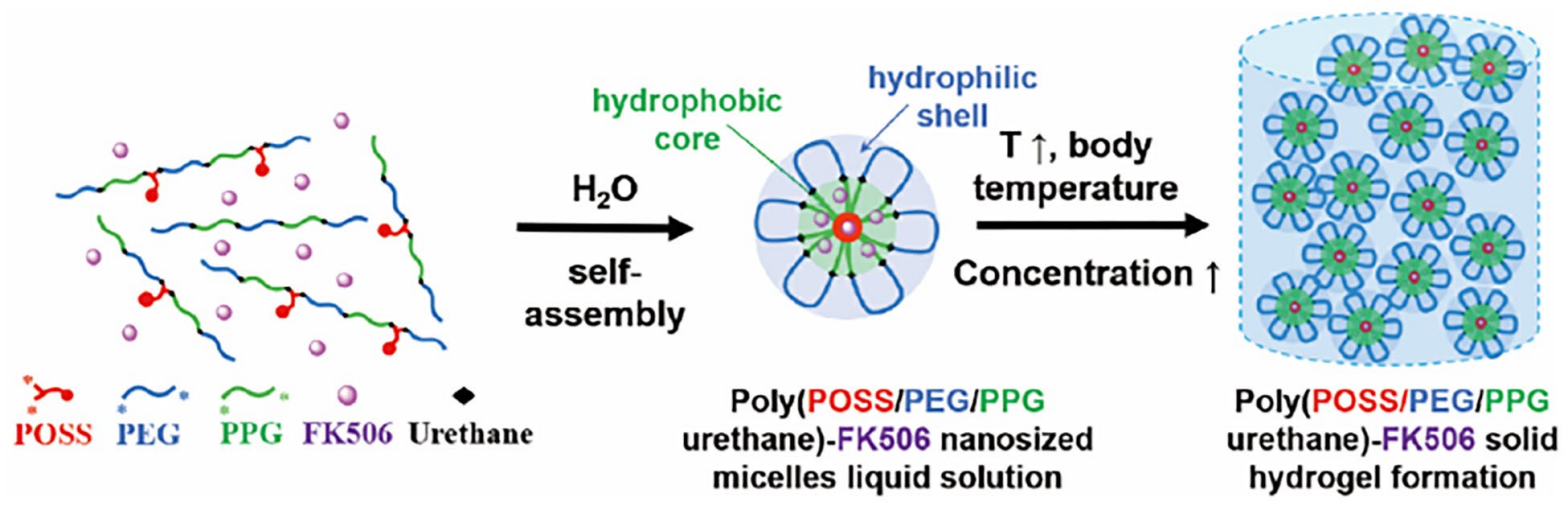
Self-assembly of MPEP and hydrogel formation in water (Han et al., 2022).Copyright 2022, Bioactive Materials.

In addition to ion-sensitive *in situ* gels, a study developed a copolymer with temperature-sensitive sol-gel transition behavior from mono-functional polyhedral oligomeric silsesquioxane (MPOSS), PEG, and polypropylene glycol (PPG) (MPOSS-PEG-PPG, MPEP) (Han et al., 2022). POSS can be incorporated physically or chemically into polymer matrices with high compatibility, resulting in enhanced thermal stability, strengthened mechanical properties, excellent optoelectronic performance, and super-hydrophobicity. Figure 5 depicts the preparation process of the self-assembly of MPEP and hydrogel formation in water. MPEP displays the appropriate micellization and gelation characteristics because of its ability to self-assemble on the ocular surface. *In vitro* and *in vivo* observations indicated that this hydrogel was biocompatible and low in toxicity. Besides, this thermo-responsive hydrogel effectively inhibited the symptom of dry eye in a C57BL/6 mice model.

## 4. Sirolimus (Rapamycin)

Sirolimus (SR), a potent immunomodulatory agent, also known as Rapamycin, is a macrolide antibiotic with potent immunosuppressive properties that act by inhibiting mTOR (Yatscoff et al., 1995, Shah, Edman et al., 2013). mTOR, an enzyme, regulates several cellular functions, including protein synthesis, lipid synthesis, cell proliferation, and activated lymphocytes (Thomson et al., 2009). As an immunosuppressant, sirolimus is commonly used in solid organ transplant rejections and has also been exploited for cancer and autoimmune diseases. In a previous study, sirolimus was reported to have a 50-fold anti-immune effect over CsA and a 30-fold effect over Tacrolimus (Benjamin et al., 2011). Unlike tacrolimus, it does not bind to calcineurin. Instead, it binds to tacrolimus-binding protein, preventing mTOR activation. Consequently, the activation, proliferation, and antibody production of T lymphocytes are inhibited as the cell cycle progresses from G1 to S phases (Murphy et al., 2011). Additionally, it inhibits inflammatory cytokine production (Paghdal & Schwartz, 2007). Ophthalmological immune disorders can be treated with this compound because sirolimus binding protein is highly expressed in corneas and retinas. By reducing the production of angiogenesis factors, inflammatory factors, and immune mediators (Kang et al., 2008), patients can avoid long-term glucocorticoid side effects. The primary disadvantage of sirolimus is its water solubility (about 2.6 μg/mL) (Simamora et al., 2001), high molecular weight (914,172) (Macdonald et al., 2000), and the absence of ionizable functional groups within a pH of 2–10. As hydrolysis and oxidation are caused by light and heat, sirolimus is unstable in aqueous media. Furthermore, sirolimus dose is closely related to its toxicity, which is reversible. The main adverse reactions are nephrotoxicity, neurotoxicity, hyperglycemia, and joint pain.

### 4.1. Microparticles

A study described sirolimus microspheres based on optimized poly (3-hydroxybutyrate-co-3-hydroxy valerate) and examined Sjögren’s syndrome model of obesity diabetes dry eye mice for its effect on the ocular surface status (Wang et al., 2019). Neither agglomerations nor adhesions were present in the microspheres, which were smooth and round with a diameter of approximately 20–50 μm. Compared with mice without any treatment, mice receiving microspheres secreted more tears and their corneal histology was normal with reduced corneal fluorescein levels. A significant improvement was observed in the signs and symptoms of tear secretion, dry eyes, histological cornea damage, and corneal endothelial cell injury in mice following sirolimus administration.

Anterior chamber (ACE) was a promising site for clinical islet transplantation (Perez et al., 2011). There are many blood vessels in the iris, which can help vascularize the transplanted islets quickly and minimize hypoxia and death. During one study, sirolimus particles and islets were transplanted into the anterior chamber in order to prevent local immune rejection of the graft. It is possible to reach the target dose of sirolimus (2.6 to 5.2 ng/day) (Abdulreda et al., 2014) through the in vitro release by mixing PCL and PLGA. In allogeneic recipients, the particles and islets were co transplanted in the right anterior chamber, while the blank particles and islets were transplanted in the left anterior chamber as a control. As a result of complete rejection, the transplanted islets could not be observed in the left eye for 17 days. It was found that the right eye remained for 30 days after transplantation, which significantly delayed rejection of the islets (Fan et al., 2019).

### 4.2. Liposomes and NLCs

According to one study, ethanol injection into sterile liposome dispersions produced sterile liposome dispersions ranging between 140 and 211 nm (Linares-Alba et al., 2016). Entrapment efficiencies between 93% and 98% were achieved. A lyophilization step was necessary to manufacture liposomes because of their poor stability in aqueous dispersion. Normal lacrimal production was duplicated after three applications of liposomes containing 1 mg/mL sirolimus. The tear film had become more stable during the treatment, which was nonexistent before the treatment.

Researchers have investigated designing sirolimus formulations using cationic lipid carriers (SR-NLCs) that are capable of loading sirolimus and protecting the drug from harsh media conditions relative to emulsions and liposomes (Zahir-Jouzdani et al., 2019). With NLCs comprising precirol ATO5 (one type of glyceride), capryol propylene glycol mono, and stearyl amine, SR-NLCs were successfully delivered to the cornea. As expected, sirolimus loaded efficiently into spherical NLCs with moderately positive surface charges. SR-NLCs downregulated transforming growth factor beta 1 (TGFβ1) and platelet-derived growth factor (PDGF) in corneal fibroblasts cell culture with angiogenesis suppression, collagen deposition, and myofibroblast formation both *in vitro* and *in vivo*. The results demonstrated that SR-NLCs were effective antifibrotic and angiogenic agents to prevent fibrosis and corneal haze after chemical burns.

### 4.3. Micelles

Three types of micelles are introduced.

1. Stable, clear, aqueous mixed nano micellar formulations (MNFs) were prepared with octoxynol-40 (Oc-40) and vitamin E TPGS as polymeric matrix components to deliver sirolimus at the back of the eye (Cholkar et al., 2015). The average size of blank MNF was 10.84 ± 0.11 nm and sirolimus-MNF was 10.98 ± 0.089 nm. Transmission electron microscope analysis revealed that micelles were spherical in shape. NMR studies conducted on MNF confirmed the absence of free sirolimus. No cytotoxicity was observed on rabbit corneal epithelial cells or human retinal pigment epithelial cells in the presence of placebo or sirolimus-loaded MNF. An *in vivo* study demonstrated that sirolimus concentrations after 1 h of single topical dosing (50 μL) in retina-choroid tissue were extremely high (362.35 ± 56.17 ng/g tissue). Based on these findings, MNF is capable of delivering sirolimus to the retina/choroid tissues after topical drop administration to the eye and it does not partition back into the vitreous humor.
2. O-octanoyl-chitosan-polyethylene glycol (OChiPEG) graft copolymers micelles prepared by thin film method were amphiphilic chitosan derivatives (Elsaid et al., 2012). Particles with a particle size of 40.6 nm and a Zeta potential of +6.84 mV exhibited high drug entrapment (85.6%) and loading efficiency (16.3%). Micelles based on OChiPEG retained sirolimus well (14.8 ± 0.81μg/g) with a high diffusion coefficient through the sclera (5.57 ± 1.04 × 10^−8^ cm^2^·s^−1^). As sirolimus was successfully permeated and retained in the scleral space, this preparation method may be useful for the topical delivery of other hydrophobic agents.
3. Researchers have used Tween 80 and P40S to prepare thin film dispersion sirolimus micelles, and prepared the sirolimus nanosuspension solution with the anti-solvent precipitation method (Wang et al., 2019). High concentration of sirolimus can be detected in rabbit eyes through HPLC-ESI-MS/MS in 3–6 hours after instillation into rabbit anterior chamber. The content of sirolimus can still be measured within 10 hours. The average C_max_ of micelle and nano suspension were 9.51 ng/mL and 23.43 ng/mL respectively (significant difference, *p* < 0.05).

### 4.4. *In situ* gel

As the charged liposomes prepared from chitosan are negatively charged, it is more likely to adhere to the charged cornea, allowing it to extend in front of the cornea and promote ocular absorption. Using both liposomes and *in situ* gels to prepare 0.2% sirolimus liposome-*in situ* gel can further prolong the drug’s residence time in the cornea (Zheng et al., 2012). It was nonirritating to the cornea of rats and has good histocompatibility. When compared to 0.2% castor oil eye drops, the peak time of *in situ* gel was twice as long as that of eye drops. The AUC of *in situ* gel was significantly higher than that of eye drops, indicating a sustained-release effect and improved bioavailability.

## 5. Everolimus

Everolimus (EV), 4 O-O- (2-hydroxyethyl)-rapamycin, a hydroxy derivative of sirolimus, is a lipophilic 31-membered ring lactone compound isolated from *Streptomyces hygroscopicus* (Kasper et al., 2018). Compared to sirolimus, everolimus has an additional 2-hydroxy-ethyl chain, making it more hydrophilic and increasing its oral bioavailability from 10% to 16% (compared with sirolimus) (Crowe et al., 1999). Additionally, compared to sirolimus, everolimus has a shorter half-life, a faster onset of steady state, and is suitable for combination therapy with CsA. Everolimus inhibits helper T cells and inhibits immune rejection (Herrera-Gómez et al., 2017) and tumor growth (Huijts et al., 2017), so it can be used for noninfectious uveitis (Li et al., 2018), autoimmune uveoretinitis, and corneal neoplasia immune-mediated rejection after corneal transplantation and angiogenesis (Blair et al., 2017).

Based on *in vitro* studies on the porcine cornea, everolimus has a much higher penetration than sirolimus, so it can replace sirolimus in immune rejection treatments (Baspinar et al., 2008).

### 5.1. Nanosuspension

Everolimus nanosuspension was prepared using P407, PVA, and hydroxypropyl methylcellulose (HPMC) as stabilizers with a size of 156.47 nm (Tang et al., 2019). Researchers have compared Tween-80 and P40S as carriers to prepare micelles of everolimus through the thin-film dispersion method. The *in vitro* release model and the rabbit scleral permeation model agreed with the Higuchi equation. In contrast to the micelles, everolimus nanosuspension exhibited a 3-fold higher AUC in pharmacokinetic studies of aqueous humor. Thus, everolimus suspension is increased bioavailability, permeability, and release rates.

### 5.2. Micelles

Micelles containing everolimus were prepared with Soluplus®, a polymer grafted from polyvinyl caprolactam, polyvinyl alcohol, and polyethylene glycol (PVCL-PVA-PEG) (Mehra et al., 2021). PEG forms the outer hydrophilic layer, while PVCL and PVA form the hydrophobic shells of the micelles to allow everolimus to penetrate the ocular barrier. An everolimus-loaded micelle was prepared via thin-film hydration/solvent evaporation. The micelle size was 65.55 nm. The micelles were characterized for surface morphology using transmission electron microscopy, scanning electron microscope, and atomic force microscope, revealing the smooth surfaces and spherical particles of the micelles. The micelles of everolimus were found to encapsulate everolimus efficiently, resulting in sustained release and significantly penetrating goat corneas effectively. Moreover, the micelles did not exhibit any ocular toxicity in the hen’s egg test on chorioallantoic membrane assay, thus the formulation can be considered safe.

Similarly, based on one polymer, mPEGhexPLA has previously been demonstrated to transport poorly soluble drugs efficiently into the anterior segment of the eye (Di Tommaso et al., 2012). After local application, mPEGhexPLA micelles were efficient at delivering drugs across biological barriers because of their inert surface properties and small size. Everolimus was efficiently dissolved in mPEGhexPLA micelles at a concentration of 5 mg/mL after encapsulation. The EV/mPEGhexPLA micelles were prepared using solvent evaporation. During murine experimental autoimmune uveoretinitis, the right eye was treated with 0.5% everolimus formulation or PBS five times a day. Compared to the PBS-treated mice, topical everolimus treatment reduced experimental autoimmune uveoretinitis severity bilaterally (Kasper et al., 2018).

### 5.3. *In situ* gel

To prepare everolimus *in situ* gel, propylene glycol was used to dissolve everolimus and then combined with a solution of PVA, poloxamer, and gellan gum. By administering 80 μL of 1 mg/mL everolimus *in situ* gel to rabbit eyes, the pharmacokinetics in rabbit aqueous humor was determined by HPLC-MS/MS. Everolimus attained its T_max_ at 3.750 ± 1.982 hours and its C_max_ was 107.256 ± 53.965 ng/mL. The AUC _(0-t)_ of the drug was 509.365 ± 172.376 ng·h/mL. The drug concentration in rabbit aqueous humor can be detected for 10 hours, indicating that the *in situ* gel can improve intraocular bioavailability and maintain a high intraocular drug concentration for a long time (Tang et al., 2019).

## 6. Patented and marketed drugs

Table 2 lists the ophthalmic preparation patents for the aforementioned immunosuppressants, whereas Table 3 lists the commercially available ophthalmic preparations. In terms of patents and marketed drugs, cyclosporine is the most common research and marketed drug whereas tacrolimus is marketed but rarely patented. Sirolimus and everolimus are two patented and unmarketed drugs. Current indications for this procedure include dry eye, rejection of corneal transplants, conjunctival xerosis, spring catarrhal conjunctivitis, and other ocular immune disorders.

**Table 2.** Patents of ophthalmic preparations related to immunosuppressants.

Drug	Dosage form	Patent Number	Stage
Cyclosporine	Particle	US11680247	In the application phase
	Emulsion	US14398012	Authorized
	Emulsion	CN201610172271.4	Authorized
	Particle	PCT/US2007/064153	In the application phase
	Film	PCT/KR2017/007426	In the application phase
	Implants	PCT/US2008/062325	In the application phase
	Emulsion-Microemulsion-Micelles	PCT/FR2016/052492	In the application phase
	Latex	JP2014203406	Authorized
	Emulsion	TW099103288	In the application phase
	Liposomes	PCT/SG2017/050216	In the application phase
Tacrolimus	Salt solvates	KR1020110096942	Authorized
	Emulsion	JP2012086806	Authorized
	Oil-based solution or ointment	US10465293	Authorized
Sirolimus	Self-emulsifying preparation	PCT/US2006/004955	In the application phase
	Suspension	US14887222	Authorized
	Self-emulsifying preparation	US11351844	In the application phase
	Micelles-suspension-*in situ* gel	CN201710557141.7	Authorized
	Inclusion complex	US13172038	Authorized
	Gelatin	US12778872	Authorized
	Liposomes	US15034098	In the application phase
	Micelles	CN201710422438.2	Authorized
	Microemulsion injection	CN201010555662.7	Authorized
Everolimus	Inclusion complex	US13172038	Authorized
	Micelles-suspension-*in situ* gel	CN201710557141.7	Authorized

Note: US, United States of America; CN, China, WO, International Bureau, JP, Japan, TW Taiwan, KR Korea.

**Table 3.** Ophthalmic preparations of immunosuppressants marketed domestically and internationally.

Product	Company	Approval Date	Drug/Dosage Form	Origin
Cyclosporine eye drops (II)	Shenyang Xingqi Eye Medicine	2021-12-30	Cyclosporine/ eye drops	China
Cequa®	SunPharmaceutical Industries	2018-08-14	Cyclosporine/ eye drops	US
Restasis®	Allergan	2003-10-10	Cyclosporine/ ophthalmic emulsion	US
Verkazia®	Santen Oy	2018-07-06	Cyclosporine/ ophthalmic emulsion	EU
Ikervis®	Santen Pharmaceutical	2015-03-19	Cyclosporine/ ophthalmic emulsion	Japan
Papilock Mini®	Santen Pharmaceutical	2006-01	Cyclosporine/ eye drops	Japan
Talymus®	Senju Pharmaceutical	2008-05	Tacrolimus/ suspension	Japan

**Figure 6.**
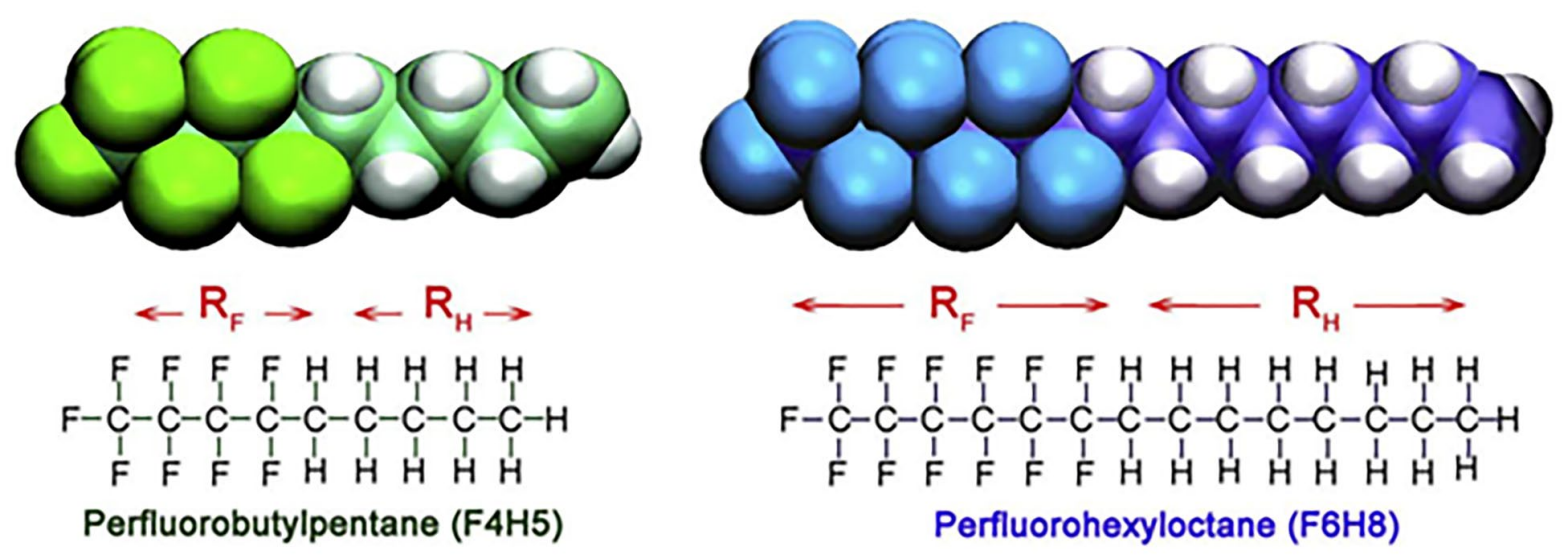
Schematic diagram of semifluorinated alkanes (Agarwal et al., 2018).Copyright 2018, International Journal of Pharmaceutics.

In addition to the aforementioned formulations, Novaliq, Heidelberg, Germany has developed CsA (Wirta et al., 2019) and tacrolimus (De Majumdar et al., 2017) eye drops based on EyeSol^TM^®, the first anhydrous drug delivery technology platform for the treatment of the dry eye. This technology mainly uses semi-fluorinated alkanes as solvents, adding insoluble drugs directly to semi-fluorinated alkanes, and adding ethanol or oily medium as a solution. Figure 6 shows that semifluorinated alkanes (Agarwal et al., 2018) consist of at least one non-fluorinated alkane segment and one perfluorinated alkane segment in which some hydrogen atoms are replaced by fluorine. Semifluorinated alkanes are straight or branched chain alkanes. It is the company’s patented product, for which dozens of related patents have been applied for. CyclASolTM—a dissolvable cyclosporine eye drop made from semifluorinated alkane 1-perfluorobutyl-pentane—is being developed by the Chinese company Hengrui.

## 7. Summary

Immunosuppressants can effectively treat some eye diseases. The high lipophilicity, low solubility, and low bioavailability of immunosuppressants, as well as the special structure of the eye, make nanodosage more clinically preferred. This article have mentioned the microparticles, nanoparticles, liposomes and NLCs, micelles, *in situ* gels of cyclosporine, tacrolimus, sirolimus (rapamycin) and everolimus. In addition, there are also some researches in cyclosporine implants, tacrolimus eye drops with the solubilizer and nanosuspensions for everolimus formulations. Most studies on immunosuppressants have focused on calmodulin inhibitors (cyclosporine and tacrolimus), whereas there are relatively few studies on mTOR inhibitors (sirolimus (rapamycin) and everolimus). mTOR inhibitors will become more popular drugs for ophthalmic immunosuppressants in the future with fewer side effects. This paper provides new strategies for future research and development of these four immunosuppressants in ophthalmic preparations.
